# Palliative cerebrospinal fluid shunting for leptomeningeal metastasis-related hydrocephalus in patients with lung adenocarcinoma: A single-center retrospective study

**DOI:** 10.1371/journal.pone.0210074

**Published:** 2019-01-10

**Authors:** Koichi Mitsuya, Yoko Nakasu, Nakamasa Hayashi, Shoichi Deguchi, Toshiaki Takahashi, Haruyasu Murakami, Tateaki Naito, Hirotsugu Kenmotsu, Akira Ono, Kazushige Wakuda, Hideyuki Harada

**Affiliations:** 1 Division of Neurosurgery, Shizuoka Cancer Center, Shizuoka, Japan; 2 Division of Thoracic Oncology, Shizuoka Cancer Center, Shizuoka, Japan; 3 Division of Radiation Oncology, Shizuoka Cancer Center, Shizuoka, Japan; University of Nebraska Medical Center, UNITED STATES

## Abstract

**Purpose:**

Management of leptomeningeal metastasis-related hydrocephalus (LM-H) is particularly challenging regarding the control of severe headache, nausea, and vomiting due to intracranial hypertension. To investigate the improvements of performance status and outcome with cerebrospinal fluid (CSF) shunt surgery for LM-H in patients with lung adenocarcinoma.

**Methods:**

Data on patients with leptomeningeal metastasis-related hydrocephalus from lung adenocarcinoma diagnosed by MR imaging and/or cytological examination were retrospectively analyzed. Between August 2008 and July 2017, the authors reviewed 31 patients requiring CSF shunt, who underwent ventriculo-peritoneal or lumbo-peritoneal shunt.

**Results:**

The patients consisted of 11 men and 20 women with a median age of 59 years. Twenty-six patients received EGFR-tyrosine kinase inhibitors (TKIs). CSF shunt surgery yielded rapid improvement in the performance status of 90.3% of patients. Median overall survival from the diagnosis of LM in patients with ECOG performance status less than 2 was 7.7 months, and this was significantly longer than those in patients with PS 3 or 4 (4.4 or 1.5 months; p<0.001). Multivariate analysis by Cox regression revealed survival differences according to PS at diagnosis of LM [PS 1–3 vs. PS4, hazard ratio (HR) 0.201, p = 0.034], controlled extracranial disease (HR 0.248, p = 0.005), and post-shunt EGFR-TKI for LM treatment (HR 0.193, p = 0.008). Postoperative symptomatic peritoneal carcinomatosis was observed in one patient (3.2%).

**Conclusion:**

CSF shunting may be a safe and effective strategy in patients with LM-H from lung adenocarcinoma. A prospective study is needed to establish the effectiveness and safety of palliative CSF shunt for LM-H.

## Introduction

Leptomeningeal metastasis (LM) is a devastating complication of cancer that affects 5% to 8% of all patients with solid tumors [1]. The median survival time of untreated patients with LM is only 4 to 6 weeks, but survival can be extended to 4 to 6 months by treatment in selected patients [2, 3].

As described previously [4], the treatment of LM usually includes intrathecal chemotherapy, systemic therapy, radiotherapy, and surgery. Surgery and radiotherapy are mainly reserved for the palliation of hydrocephalus or symptoms resulting from focal lesions [5–8].

Systemic chemotherapy is a main option to treat systemic disease, but data on its effect for patients with LM are lacking. Although a few recent case series on epidermal growth factor receptor-tyrosine kinase inhibitor (EGFR-TKI) use for patients with LM have been reported, these studies included only a small number of patients [4, 9, 10].

As described in detail previously [11], without cerebrospinal fluid (CSF) diversion, patients with hydrocephalus may develop severe headaches, and cognitive, gait, or other neurological deficits, sometimes leading to death. CSF diversion techniques such as ventriculo-peritoneal (VP) or lumbo-peritoneal (LP) shunting are safe and effective in treating hydrocephalus, but these techniques have not been used extensively in patients with LM due to concerns about the peritoneal cancer dissemination [12, 13].

We investigated the outcomes of CSF shunting and concomitant multimodal therapy for LM with hydrocephalus (LM-H) from lung adenocarcinoma in our institution.

## Materials and methods

### Participants and recruitment

Approved for this study was obtained from the institutional research ethics board of Shizuoka Cancer Center (28-J173-28-1-3). The requirement for written informed consent was waived. We searched the electronic database of the division of neurosurgery at our institution, all data were fully anonymized before assessment. We reviewed 31 patients with LM-H from lung adenocarcinoma requiring CSF shunting between August 2008 and July 2017. The patients with LM diagnosed by MR imaging and cytological examination. CSF shunting followed by targeted therapy and/or radiotherapy was indicated when a patient had controlled extra-central nervous system (CNS) metastases and systemic life expectancy longer than three months after the control of LM-H. The patients underwent either VP (n = 13) or LP (n = 18) shunting for hydrocephalus. An adjustable valve (Strata Valve or Strata NSC L/P Valve; Medtronic PS Medical, Minneapolis, MN, USA) was used with the shunt system (peritoneal catheter and ventricular catheter or lumbar catheter; Medtronic PS Medical) for all cases.

This study did not include patients treated with IT chemotherapy. Because this study cohort is from 2008 to 2017, in the era of EGFR-TKI, we expected that EGFR-TKI is more effective for leptomeningeal metastasis than IT chemotherapy for EGFR mutant population.

### Data collection

We reviewed the patients’ charts for their characteristics, indications for a CSF shunt, shunt procedures, and complications. Survival was analyzed using Kaplan-Meier estimates; potential prognostic factors were evaluated using Cox proportional hazards model. Statistical analysis was performed using JMP (version 11; SAS Institute).

## Results

**Fig 1:**
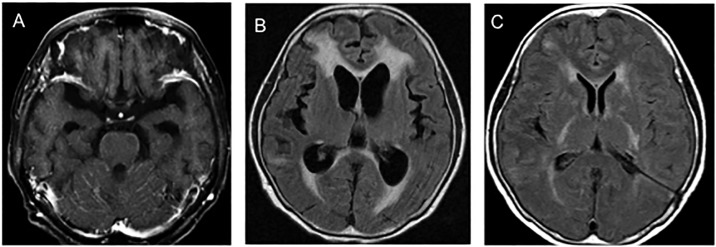
MR image before and after shunt surgery. (A) MR image shows linear enhancement along the cerebellar folia. (B) FLAIR image obtained prior to VP shunt shows enlarged lateral ventricles with periventricular high intensity. (C) FLAIR image after VP shunt shows decreased size of the lateral ventricles and improved periventricular high intensity three months after shunting.

The patients consisted of 11 men and 20 women with a median age at LM-H of 59 years (range 36–76). Twenty-one patients had EGFR mutation and five patients EGFR wild-type NSCLC (Table 1). The other five were non-smoking female patients. Twenty-six patients received EGFR-TKI (gefitinib, erlotinib, or osimertinib). Progressive and symptomatic communicating hydrocephalus presented in all patients (Fig 1).

**Table 1:** Characteristics of patients with CSF shunting for LM-H from lung adenocarcinoma.

	EGFR mutant number (%) (n = 26)	EGFR wild type number (%) (n = 5)	All number (%) (n = 31)
Age: median, years	59	66	59
Sex			
Male	7 (27)	4 (80)	11 (37)
Female	19 (73)	1 (20)	20 (63)
EGFR mutation state			
19del/21L858R/21L861Q/non-Sm+F	10 (42)/10 (42)/1 (4)/5 (19)	wild type	
ALK fusion protein	negative	negative	
**Systemic therapy**			
Before LM			
Gefitinib	11 (42)	0	11 (35)
Erlotinib	10 (42)	0	10 (32)
Osimertinib	2 (8)	0	2 (6)
Chemotherapy	1 (4)	5 (100)	6 (19)
None	2 (8)	0	2 (6)
After LM			
Gefitinib	3 (12)	0	3 (10)
Erlotinib	15 (58)	0	15 (48)
Osimertinib	3 (12)	0	3 (10)
Chemotherapy	1 (4)	0	1 (3)
None	4 (15)	5 (100)	9 (29)
**Radiation therapy**			
Before LM			
WBRT for BM	9 (35)	0	9 (29)
After LM			
WBRT	9 (35)	2 (40)	11 (35)
CSI	4 (15)	1 (20)	5 (16)
No RT	4 (15)	2 (40)	6 (19)
**ECOG PS**			
at diagnosis of LM: PS1/2/3/4	2/8/14/2	0/0/3/2	2/8/17/4
Before shunt: PS1/2/3/4	0/2/17/7	0/0/2/3	0/2/19/10
After shunt: PS 1/2/3/4	5/14/6/1	1/2/1/1	6/16/7/2

Gef, gefitinib; Erl, erlotinib; Osim, osimertinib; NA, not analysis; Non Smok/Fem, non-smoker female patient; WBRT, whole-brain radiotherapy; BM, brain metastasis; CSI, cranio-spinal irradiation; PS, performance status

Median overall survival (OS) was 4.5 months from the onset of LM and 3.5 months after CSF shunt. Those with the longest survival were alive 15 months from the diagnosis of LM-H. Five patients (5/31; 16%) was long term survival over 300 days from diagnosis of leptomeningeal metastasis (S1 Table). These patients were all EGFR mutation positive.

**Fig 2:**
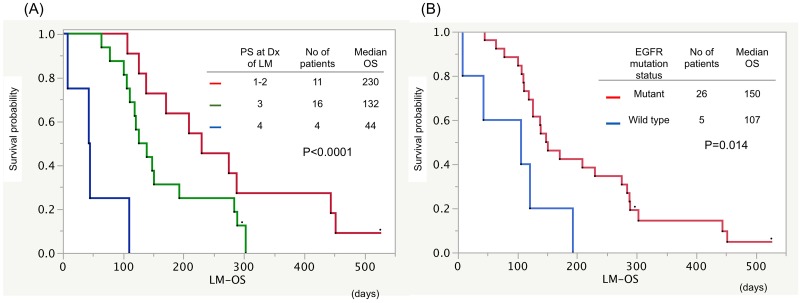
Kaplan-Meier survival curves showing OS from the diagnosis of LM (LM-OS). (A) Median OS from the diagnosis of LM-H (LM-OS) in patients with ECOG performance status (PS) less than 2 at the diagnosis of LM was 7.7 months, and this was significantly longer than those in patients with PS 3 or 4 (4.4 or 1.5 months, respectively; p<0.001). (B) Patients with EGFR sensitive mutation showed longer LM-OS than those with EGFR wild type (5.0 vs. 3.6 months, respectively; p = 0.019).

**Fig 3:**
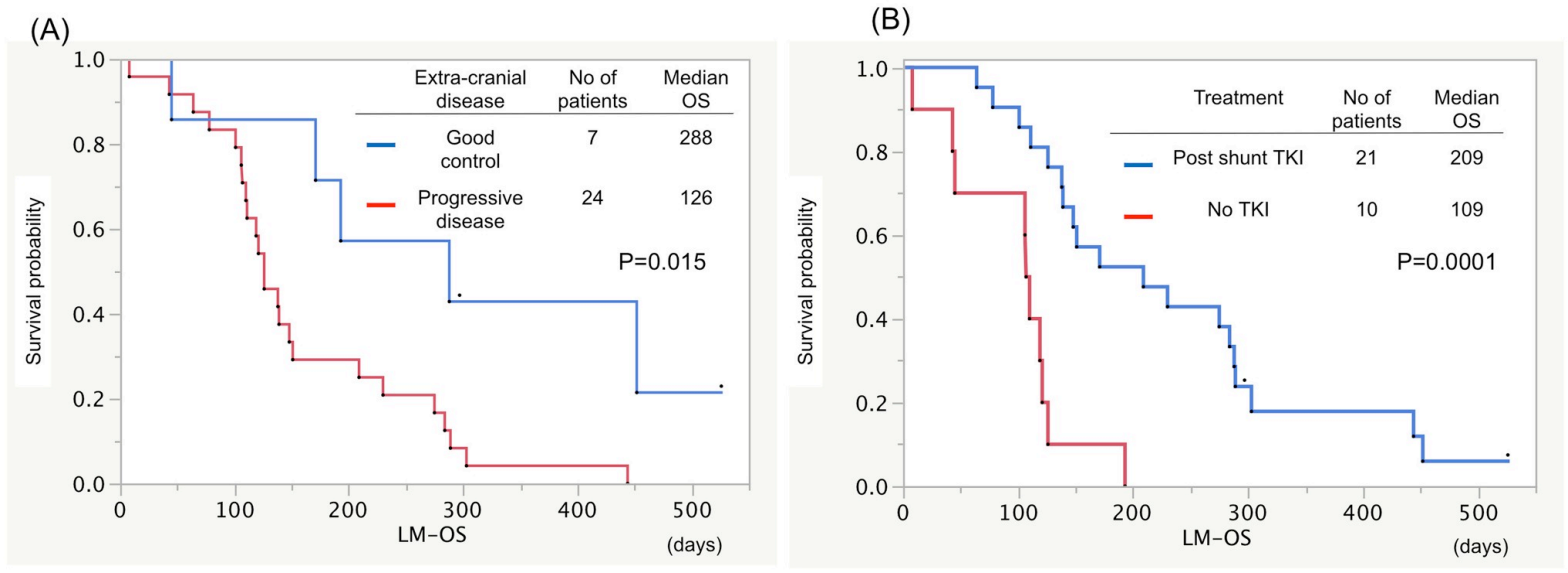
Kaplan-Meier survival curves showing OS from the diagnosis of LM (LM-OS). (A) Patients with good control of extracranial disease showed prolonged LM-OS compared with the progressive extracranial disease group (9.6 vs. 4.2 months, respectively; p = 0.015). (B) Patients receiving systemic EGFR-TKI treatment after CSF shunt showed prolonged LM-OS compared with the no TKI group (7.0 vs. 3.6 months, respectively; p = 0.0001).

**Fig 4:**
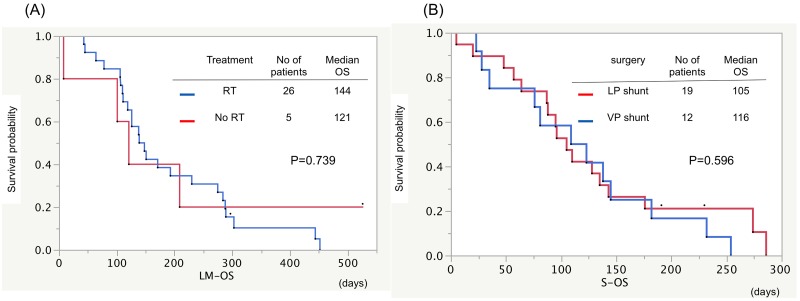
Kaplan-Meier survival curves showing OS from the diagnosis of LM (LM-OS) (a) and OS from CSF shunting (S-OS) (b). (A) There was no significant difference in LM-OS between patients who did or did not receive radiotherapy (4.0 vs. 3.5 months, respectively; p = 0.739). (B) There was no significant difference in OS from shunt surgery between patients who underwent VP shunt and LP shunt procedures (3.9 vs 3.5 months, respectively; p = 0.596).

**Fig 5:**
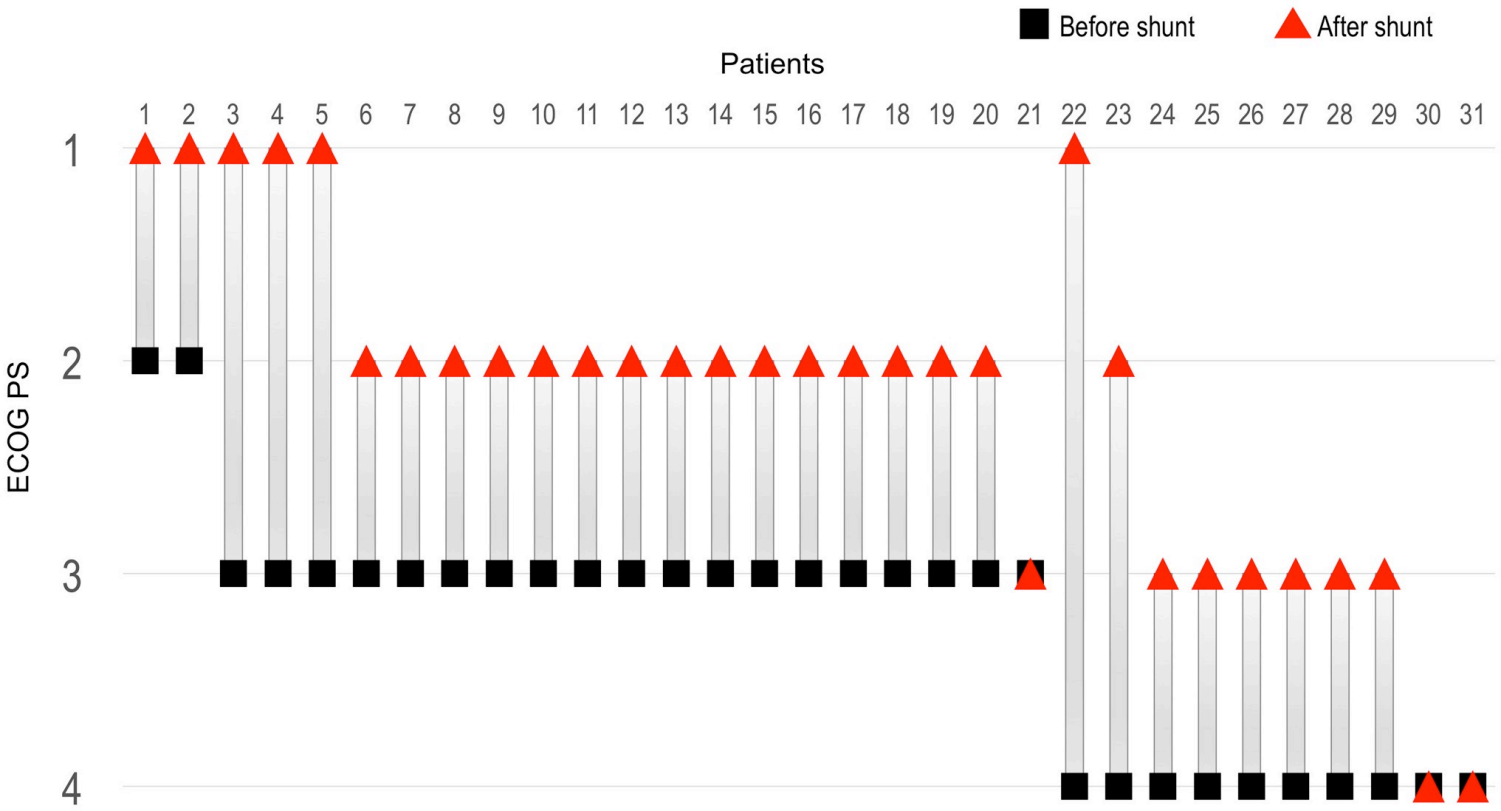
ECOG performance status comparison before vs. after shunt surgery. Remarkable improvements in ECOG PS after shunt surgery.

Median OS from the diagnosis of LM-H (LM-OS) in patients with ECOG performance status (PS) less than 2 at the diagnosis of LM was 7.7 months, and this was significantly longer than those in patients with PS 3 or 4 (4.4 or 1.5 months, respectively; p<0.001, Fig 2A). Patients with EGFR sensitive mutation showed longer LM-OS than those with EGFR wild type (5.0 vs. 3.6 months, respectively; p = 0.019, Fig 2B). Patients with good control of extracranial disease showed prolonged LM-OS compared with the progressive extracranial disease group (9.6 vs. 4.2 months, respectively; p = 0.015, Fig 3A). The patients with EGFR-TKI therapy after shunting had significantly longer LM-OS than the patients with other treatment did (7.0 vs. 3.6 months, respectively; p = 0.003, Fig 3B). No significant difference of median LM-OS was observed between those with or without radiation therapy (4.8 vs. 4.0 months, p = 0.739, Fig 4A). Moreover, no significant difference of median OS from surgery (S-OS) was observed between patients undergone VP and LP shunt procedures (3.9 vs. 3.5 months; p = 0.880, Fig 4B). CSF shunt surgery yielded rapid improvement in the performance status of 90.3% of patients (Fig 5).

Multivariate analysis using a Cox regression hazard model revealed that there were survival differences according to PS at diagnosis of LM-H (PS 1–3 vs. PS4, HR 0.201, 95% CI 0.048–0.880, p = 0.034), controlled extracranial disease (HR 0.248, 95% CI 0.070–0.679, p = 0.005), and post-shunt EGFR-TKI (HR 0.193, 95% CI 0.060–0.632, p = 0.008) (Table 2).

**Table 2:** Cox regression analysis of potential prognostic factors.

Variable	HR	95% CI	p value
ECOG PS at diagnosis of LM			
PS 1–3 vs. 4	0.201	0.048–0.880	0.034
Controlled extracranial disease			
Yes vs. No	0.249	0.070–0.679	0.005
Post-shunt EGFR TKI			
Yes vs. No	0.193	0.060–0.632	0.008

### Adverse events of CSF shunting for LM-H

One patient (3.2%) suffered from peritoneal cancer dissemination that we thought not related to shunt procedure, but likely to be the progression of the systemic cancer. Infection occurred in two patients (6.5%) after VP shunt. One patient suffered from catheter disposition hat needed a revision surgery after VP shunt. And another presented low intracranial pressure that was immediately controlled by alternating valve pressure after LP shunt. There were no instances of obstructive shunt malfunction.

## Discussion

LM is one of the most devastating complications and remains a serious concern in the clinical course of lung adenocarcinoma. The survival time of lung adenocarcinoma patients affected by LM is approximately 3 months, which is shorter than that of patients with LM from other cancers, such as breast cancer and hematological malignancies [14–16]. More than half of patients die of leptomeningeal progression once they develop LM from lung adenocarcinoma [17]. Management of LM-H is particularly challenging regarding the control of severe symptoms due to intracranial hypertension.

### Main findings of the study

In this study, the median overall survival of the patients after the diagnosis of LM-H was 4.5 months and survival after shunt surgery was 3.5 months. Omuro et al. reported on VP shunt for LM-H in 37 patients, with improvement of symptoms being achieved in 77% despite the relatively short median survival of 2 months after shunt [18]. In addition, Jung et al. reported that surgically untreated LM-H showed poor overall survival compared with surgically treated hydrocephalus (1.7 vs. 5.7 months, respectively; no statistical significance) [19]. These two reported studies included patients with LM from lung, breast, and various other cancers.

Our study showed that patients who received TKI therapy after shunt surgery had significantly longer LM-OS than patients without TKI therapy (7.0 vs. 3.6 months, respectively; p = 0.001). Multivariate analysis revealed that there were survival differences according to PS at the diagnosis of LM (PS 1–3 vs. PS4, HR 0.201, p = 0.034), controlled extracranial disease (HR 0.248, p = 0.005), and post-shunt EGFR-TKI for LM treatment (HR 0.193, p = 0.0078).

Several studies have reported that EGFR-TKIs are a potential treatment option for patients with LM from NSCLC [4, 20, 21]. Li et al. reported that the median OS of patients after the diagnosis of LM was 8.3 months, and 88 patients who received TKIs after LM demonstrated significantly longer survival than those who did not (10.0 vs. 3.3 months) [22]. Gong et al. reported that icotinib might be effective for LM in NSCLC with EGFR sensitive mutation. The authors reported that the median overall survival from the diagnosis of LM was 10.1 months [23]. These findings indicate that EGFR-TKIs can effectively control LM from NSCLC in patients with an EGFR mutation.

The present study showed good PS at the diagnosis of LM as a significant prognostic factor regarding LM-OS (Fig 2A). Careful watching and screening are important, since patients with EGFR mutation tend to suffer from LM [24] and require its early detection by clinical assessment and brain MR imaging. Similar to our findings, Lee et al. reported that a poor PS score at the diagnosis of LM was a poor prognostic factor in 149 lung cancer patients with LM [4]. However, several previous studies indicated that age, a poor PS score, time between primary tumor and LM diagnosis of 12 months or less, and coexistent bulky metastatic disease in the CNS were negative prognostic factors [14, 16, 25]. But these findings should be carefully adapted because of the small sample size included in this study.

Intrathecal chemotherapy with methotrexate, cytarabine (DepoCyte), and thioTEPA has usually been used for the treatment of LM, however, no clear evidence demonstrating that it confers a survival advantage [26, 27]. As for WBRT, there is no consensus regarding whether it has a survival benefit [4, 28, 29]. Thus, the effects of IT chemotherapy and WBRT remain controversial.

Our results of implicated that three factors were associated with better outcome after shunt surgery: treatment with TKIs, good PS, and controlled extracranial cancer. This supports that patients treated with shunt for LM-H have similar survival outcomes and similar clinical factors of improved survival concordant with previous studies of survival in LM. From the present study, we concluded these three criteria may become an optimal indication for CSF shunting for patients with LM-H.

### Strengths and limitations

We demonstrated that patients’ PS rapidly improved after shunt surgery and they recovered to a state matching the indication criteria for EGFR-TKI administration for lung adenocarcinoma. CSF shunt is a powerful tool against severe headache and uncontrollable intracranial hypertension, and leads to a survival benefit with EGFR-TKI therapy following shunt surgery.

Lumbo-peritoneal shunting may be a more effective method for a poor condition because it is less invasive than ventriculo-peritoneal shunting, due to the short operation time and the wound being outside of the field in whole-brain radiotherapy [30, 31].

There are several limitations to this study. First, this was a retrospective study of therapeutic outcome, so patient selection bias remained a potential source of error. Second, because of the retrospective nature of this study, the treatment regimen was selected for each individual case. Third, the number of patients was relatively small.

## Conclusion

In conclusion, our data demonstrate that CSF shunting may effectively control intracranial hypertension due to LM-H from lung adenocarcinoma in patients with an EGFR mutation, especially for patients with a good ECOG PS and controlled extracranial cancer. A prospective study is necessary to establish the efficiency of CSF shunting and targeted therapy for LM-H from lung adenocarcinoma with an EGFR sensitive mutation.

## Supporting information

S1 Table Patient characteristics and treatment outcome. (DOCX)
