# Supplementary material for: Palliative cerebrospinal fluid shunting for leptomeningeal metastasis-related hydrocephalus in patients with lung adenocarcinoma: A single-center retrospective study
Source: PLoS One. 2019 Jan 10;14(1):e0210074. doi: 10.1371/journal.pone.0210074 (PMC6328154; doi:10.1371/journal.pone.0210074)
Supplement: S1 Table — (DOCX) [file pone.0210074.s001.docx]

| No. | Age | Sex | EGFR mutation | Before LM-TKI | After LM-TKI | After LM CTx | RT | PS at Dx LM | PS before shunt | PS after shunt | Time from Dx LM | Time from shunt | only CNS metastasis | Shunt procedure | CSF cell count | CSF protein | Cause of death | Adverse event after shunt | Disease special feature |
| --- | --- | --- | --- | --- | --- | --- | --- | --- | --- | --- | --- | --- | --- | --- | --- | --- | --- | --- | --- |
| 1 | 71 | F | 21L858R | Erl | Erl | − | − | 3 | 3 | 2 | 526- | 191- | − | LP | 4 | 25 | (alive) | Low intracranial pressure |  |
| 2 | 51 | F | 19 del | Erl | − | DTX+Ram | previous RT | 4 | 3 | 2 | 110 | 48 | − | LP | 4 | 84 | Systemic |  |  |
| 3 | 67 | M | 19 del | Erl | − | − | RT | 3 | 3 | 2 | 119 | 105 | − | LP | NA | NA | CNS |  |  |
| 4 | 68 | F | 21L858R | Erl | Erl | − | previous RT | 4 | 4 | 4 | 45 | 20 | Yes | LP | 4 | 348 | CNS |  |  |
| 5 | 72 | M | 21L858R | Osim | Osim | − | − | 3 | 4 | 3 | 101 | 88 | − | LP | 6 | 141 | CNS |  |  |
| 6 | 45 | F | 19 del | Osim | Osim | − | previous RT | 3 | 3 | 2 | 445- | 388- | Yes | LP | 35 | 80 | (alive) |  |  |
| 7 | 52 | F | 19 del | Erl | Erl | − | RT | 2 | 2 | 1 | 209 | 95 | − | LP | 1 | 124 | CNS |  |  |
| 8 | 42 | F | 19 del | Erl | Osim | − | previous RT | 3 | 2 | 1 | 151 | 143 | − | LP | 1 | 52 | Systemic |  |  |
| 9 | 44 | M | 21L858R | Erl | Erl | Nivo | RT | 2 | 3 | 1 | 126 | 110 | − | LP | 3 | 43 | Systemic | peritoneal dissemination | malignant pluiral and pericardial effusion at Dx of lung cancer |
| 10 | 63 | F | 21L858R | Erl | Erl | − | previous RT | 3 | 3 | 1 | 303 | 274 | − | LP | 4 | 43 | CNS |  |  |
| 11 | 61 | F | 19 del | Erl | Erl | − | RT | 3 | 3 | 1 | 139 | 135 | − | LP | 3 | 20 | CNS |  |  |
| 12 | 55 | F | 19 del | Gef | Erl | − | previous RT | 2 | 3 | 2 | 138 | 128 | − | LP | 12 | 136 | CNS |  |  |
| 13 | 59 | F | 21L858R | Gef | Erl | − | previous RT | 3 | 3 | 2 | 289 | 286 | − | LP | 8 | 96 | CNS |  |  |
| 14 | 66 | F | 21L858R | Erl | Erl | − | previous RT | 3 | 4 | 3 | 78 | 64 | − | LP | 1 | 161 | CNS |  |  |
| 15 | 71 | F | 19 del | Gef | − | − | RT | 3 | 4 | 3 | 126 | 96 | − | LP | 0 | 33 | CNS |  |  |
| 16 | 70 | F | 21L858R | Gef | Erl | − | RT | 3 | 3 | 2 | 111 | 109 | − | VP | 2 | 37 | CNS |  |  |
| 17 | 53 | M | 19 del | Erl | Erl | Bev | RT | 2 | 3 | 2 | 171 | 138 | Yes | VP | 1 | 7 | CNS |  |  |
| 18 | 73 | M | 21L858R | − | Gef | − | − | 1 | 3 | 2 | 452 | 23 | Yes | VP | 4 | 87 | CNS |  |  |
| 19 | 59 | M | 19 del | − | Gef | − | RT | 3 | 3 | 3 | 284 | 35 | − | VP | 6 | 22 | CNS | ventricular catheter disposition, infection | skin folliculitis from EGFR-TKI |
| 20 | 50 | F | 21L858R | Gef | − | − | RT | 2 | 4 | 3 | 107 | 76 | − | VP | 0 | 13 | CNS |  |  |
| 21 | 57 | M | 21L861Q | Gef | − | − | − | 3 | 3 | 2 | 64 | 57 | − | LP | 4 | 39 | CNS |  |  |
| 22 | 39 | F | Ad/F/NoSm | Gef | Erl | − | − | 3 | 4 | 3 | 148 | 145 | − | VP | 1 | 2 | CNS | infection | bacterial meningitis |
| 23 | 52 | F | Ad/F/NoSm | Gef | Erl | − | RT | 1 | 3 | 2 | 444 | 81 | − | VP | 13 | 27 | CNS |  |  |
| 24 | 60 | F | Ad/F/NoSm | − | Erl | − | RT | 2 | 3 | 2 | 275 | 254 | − | VP | 5 | 8 | CNS |  |  |
| 25 | 58 | F | Ad/F/NoSm | Gef | Erl | − | previous RT | 2 | 4 | 2 | 230 | 182 | − | VP | 0 | 6 | CNS |  |  |
| 26 | 72 | F | Ad/F/NoSm | Gef | Gef | − | RT | 2 | 3 | 2 | 288 | 232 | Yes | VP | 18 | 24 | CNS |  |  |
| 27 | 66 | M | Wt | − | − | − | RT | 3 | 3 | 2 | 106 | 87 | − | LP | 75 | 29 | CNS |  |  |
| 28 | 53 | M | Wt | − | − | − | RT | 3 | 4 | 1 | 193 | 176 | Yes | LP | 36 | 58 | CNS |  |  |
| 29 | 36 | M | Wt | − | − | − | − | 4 | 4 | 3 | 8 | 5 | − | LP | 14 | 48 | CNS |  |  |
| 30 | 71 | M | Wt | − | − | − | − | 3 | 3 | 2 | 121 | 123 | − | VP | 8 | 43 | CNS |  |  |
| 31 | 76 | F | Wt | − | − | − | RT | 4 | 4 | 4 | 43 | 28 | − | VP | 15 | 22 | CNS |  |  |

Abbreviations: CTx, chemotherapy; PS, performance status; Dx, diagnosis; Ad, adenocarcinoma; NoSm, Non-smoker; ECOG, Eastern Clinical Oncology Group; Gef, Gefitinib; Erl, Erlotinib; Osim, Osimertinib; RT, radiation therapy; Wt, wild type; DTX, docetaxel; Ram, Ramucirumab; Nivo, Nivolumab; Bev, Bevacizumab; CSDH, chronic subdural hematoma
